# PARP inhibition by olaparib or gene knockout blocks asthma-like manifestation in mice by modulating CD4^+^ T cell function

**DOI:** 10.1186/s12967-015-0583-0

**Published:** 2015-07-14

**Authors:** Mohamed A Ghonim, Kusma Pyakurel, Salome V Ibba, Amir A Al-Khami, Jeffrey Wang, Paulo Rodriguez, Hamada F Rady, Ali H El-Bahrawy, Matthew R Lammi, Moselhy S Mansy, Kamel Al-Ghareeb, Alistair Ramsay, Augusto Ochoa, Amarjit S Naura, A Hamid Boulares

**Affiliations:** The Stanley Scott Cancer Center, School of Medicine, Louisiana State University Health Sciences Center, 1700 Tulane Ave, New Orleans, LA 70112 USA; Microbiology and Immunology Department, Faculty of Pharmacy, Al-Azhar University, Cairo, Egypt; Department of Zoology, Faculty of Science, Tanta University, Tanta, Egypt; Department of Microbiology, Immunology and Parasitology, Louisiana State University Health Sciences Center, New Orleans, LA USA; Pulmonary and Critical Care Section, School of Medicine, Louisiana State University, New Orleans, LA USA; Department of Biochemistry, Panjab University, Chandigarh, India

**Keywords:** PARP inhibition, Olaparib (AZD2281), Allergen-induced eosinophilia, Adoptive transfer, Th2 cytokines, Allergen-specific IgE

## Abstract

**Background:**

An important portion of asthmatics do not respond to current therapies. Thus, the need for new therapeutic drugs is urgent. We have demonstrated a critical role for PARP in experimental asthma. Olaparib, a PARP inhibitor, was recently introduced in clinical trials against cancer. The objective of the present study was to examine the efficacy of olaparib in blocking established allergic airway inflammation and hyperresponsiveness similar to those observed in human asthma in animal models of the disease.

**Methods:**

We used ovalbumin (OVA)-based mouse models of asthma and primary CD4^+^ T cells. C57BL/6J WT or PARP-1^−/−^ mice were subjected to OVA sensitization followed by a single or multiple challenges to aerosolized OVA or left unchallenged. WT mice were administered, *i.p.*, 1 mg/kg, 5 or 10 mg/kg of olaparib or saline 30 min after each OVA challenge.

**Results:**

Administration of olaparib in mice 30 min post-challenge promoted a robust reduction in airway eosinophilia, mucus production and hyperresponsiveness even after repeated challenges with ovalbumin. The protective effects of olaparib were linked to a suppression of Th2 cytokines eotaxin, IL-4, IL-5, IL-6, IL-13, and M-CSF, and ovalbumin-specific IgE with an increase in the Th1 cytokine IFN-γ. These traits were associated with a decrease in splenic CD4^+^ T cells and concomitant increase in T-regulatory cells. The aforementioned traits conferred by olaparib administration were consistent with those observed in OVA-challenged PARP-1^−/−^ mice. Adoptive transfer of Th2-skewed OT-II-WT CD4^+^ T cells reversed the Th2 cytokines IL-4, IL-5, and IL-10, the chemokine GM-CSF, the Th1 cytokines IL-2 and IFN-γ, and ovalbumin-specific IgE production in ovalbumin-challenged PARP-1^−/−^mice suggesting a role for PARP-1 in CD4^+^ T but not B cells. In ex vivo studies, PARP inhibition by olaparib or PARP-1 gene knockout markedly reduced CD3/CD28-stimulated *gata*-*3* and *il4* expression in Th2-skewed CD4^+^ T cells while causing a moderate elevation in *t*-*bet* and *ifn*-*γ* expression in Th1-skewed CD4^+^ T cells.

**Conclusions:**

Our findings show the potential of PARP inhibition as a viable therapeutic strategy and olaparib as a likely candidate to be tested in human asthma clinical trials.

## Background

Contrary to a number of chronic diseases, asthma incidence is on the rise [1]. In the United States alone, more than 20 million individuals suffer from the disease. A sizable portion of these asthmatics do not respond to the existing drugs [2]. Accordingly, the need for new drugs as mono or adjuvant therapies is immediate.

The pathogenesis of asthma involves several cellular and non-cellular factors including Th2 and Th17 CD4^+^ T cells as well as B cells in addition to circulating factors such as IL-4, IL-5, IL-13 and many others [3]. Targeting the function of these cells and the ensuing production of Th2 cytokines and IgE has been a critical objective both in the clinic and in the laboratory. Our laboratory pioneered the studies demonstrating the involvement of poly(ADP-ribose)polymerase (PARP)-1 in asthma [4–8]. Our studies as well as those of others [9–13] suggest that the protein may constitute a viable target for the treatment of the disease. PARP-1, a member of a large family of proteins, is a DNA repair-associated enzyme that participates in the recruitment and trafficking processes of DNA repair proteins and histones to the DNA lesions primarily through base excision repair [14]. However, our laboratory and many others have suggested a role for the enzyme in a number of inflammatory conditions and regulation of transcription. We have shown that it controls NF-κB nuclear trafficking and thus transcription of NF-κB-dependent genes including those critical for asthma manifestation [15–17]. We have also shown that PARP-1 controls the fate of STAT-6 upon IL-4 or allergen exposure both in vitro and in an animal model of the disease through a calpain-dependent mechanism [8].

An ultimate goal of our studies is to explore the possibility that PARP can be targeted for therapy to treat asthma in human subjects. A great deal of effort has been made to generate potent inhibitors of the enzyme targeting cancer and inflammatory diseases [18]. Recently, olaparib (AZD2281), a small molecule inhibitor of PARP-1 and PARP-2 showed great potential for the treatment of BRCA-negative breast and ovarian cancer [19]. These neoplastic conditions were specifically targeted because the cancer cells accumulate fatal dsDNA breaks when exposed to DNA damaging agents in the absence of PARP activity leading a synthetic lethality phenotype [20]. Because this process occurs only in BRCA-mutant cancer cells, PARP inhibition is not expected to affect normal cells. In several clinical trials, the drug showed a remarkable therapeutic efficacy with an acceptable safety index in cancer patients [21]. It is noteworthy that other PARP inhibitors have also been developed and are currently tested in more than 20 clinical trials.

In the current study, we aimed to test the efficacy of olaparib in experimental asthma. We specifically examined whether olaparib administration at doses that can be translated to human therapy blocks some or all asthma-like traits. We also examined whether the drug blocks already established disease to mimic what actually occurs in human asthmatics.

## Methods

### Animals

C57Bl/6J wild type (WT) and OT-II mice (6–8 weeks old) were purchased from Jackson Laboratories (Bar Harbor, ME, USA). C57BL/6 PARP-1^−/−^ mice were generated through a backcrossing with C57BL/6 WT mice for eleven generations. The last generation was interbred to generate the C57BL/6 PARP-1^−/−^ mice. WT mice generated through the PARP-1^+/−^ mice breeding were also included in the experiment. Mice were bred in a specific-pathogen free facility at LSUHSC, New Orleans, LA, and allowed unlimited access to sterilized chow and water. Maintenance, experimental protocols, and procedures were approved by the LSUHSC Animal Care & Use Committee.

### Ovalbumin (OVA) sensitization and challenge, Airway hyper responsiveness (AHR), organ recovery, staining, Th2 cytokine and IgE assessments, and FACS analysis

Mice were sensitized to chicken OVA (Sigma-Aldrich, St. Louis, MO, USA) as described [6]. The mice were then challenged with aerosolized OVA for 30 min once (single challenge) or once a day for 3 days (multiple challenge). Control groups were not sensitized or challenged. Additional groups of mice received *i.p.* 1, 5, or 10 mg/kg olaparib (Selleckchem, Pittsburgh, PA, USA) in saline 30 min after OVA challenge. AHR, organ recovery, histopathology, bronchoalveolar lavage (BAL), cytokine and OVA-specific IgE assessment, and FACS analysis were performed as described [6, 22, 23]. To determine CD4^+^ T cell populations, spleens were processed to generate single cell suspensions after which splenocytes were stained with antibodies to mouse CD3e (145-2c11-APC) and CD4-FITC (clone RM4-5) (both from e-Bioscience, San Diego, CA, USA). To determine T-regulatory (T-reg) cell populations, splenocytes were stained with CD4 (GK1.5-FITC) and CD25-APC (clone PC61) (from Biolegend, San Diego, CA, USA), and intracellularly with anti-mouse Foxp3 (FJK-16s)-PE (e-Bioscience) followed by FACS analysis. The multiplex assay and FACS were conducted at the LSUHSC Comprehensive Alcohol Research Center Core.

### CD4^+^ T cell purification, Th1/Th2 skewing, TCR stimulation, Adoptive transfer, and RT-PCR

OT-II or WT mice were sacrificed and splenic CD4^+^ T cells were isolated by negative selection (Stem Cell Technologies, Vancouver, Canada). Purified CD4^+^ T cells were stimulated on coated plates with antibodies to CD3 (1 μg/ml) and CD28 (0.5 μg/ml) (e-bioscience, San Diego, CA, USA) then skewed toward a Th1 or Th2 phenotype as described [23]. WT CD4^+^ T cells were skewed in the absence or presence of 5 μM olaparib. RNA was extracted using Qiagen RNA extraction kit according to the manufacturer instructions. The extracted total RNA was used for the generation of cDNA using reverse transcriptase III (Invitrogen) and quantitative PCR was conducted using primer sets (IDT, San Jose, CA, USA) specific for mouse *gata*-*3*, *il*-*4*, *t*-*bet*, *ifn*-*γ*, or *β*-*actin* as described [23, 24]. Quantitative determination of gene expression levels using a 2-step cycling protocol was conducted on a MyIQ Cycler (Bio-Rad, Hercules, CA, USA). Relative expression levels were calculated using the 2[−Delta Delta C(T)] method [25]. Quantities of all targets were normalized to the mouse β-actin gene.

Th2-like cells from OT-II mice were administered *i.v*. into the tail vein of recipient mice (1 × 10^6^ cells/mouse). All mice were subjected to OVA challenge daily for 4 days. Mice were sacrificed 48 h after the last challenge.

### Data analysis

All data are expressed as means ± SEM of values from at least five mice per group unless stated otherwise. PRISM software (GraphPad, San Diego, CA, USA) was used to analyze the differences between experimental groups by one way analysis of variance (ANOVA) followed by Tukey’s multiple comparison test.

## Results

### Olaparib blocks airway eosinophilia, mucus and IgE production, and AHR upon a single or repeated challenge with OVA in a mouse model of asthma

**Figure 1:**
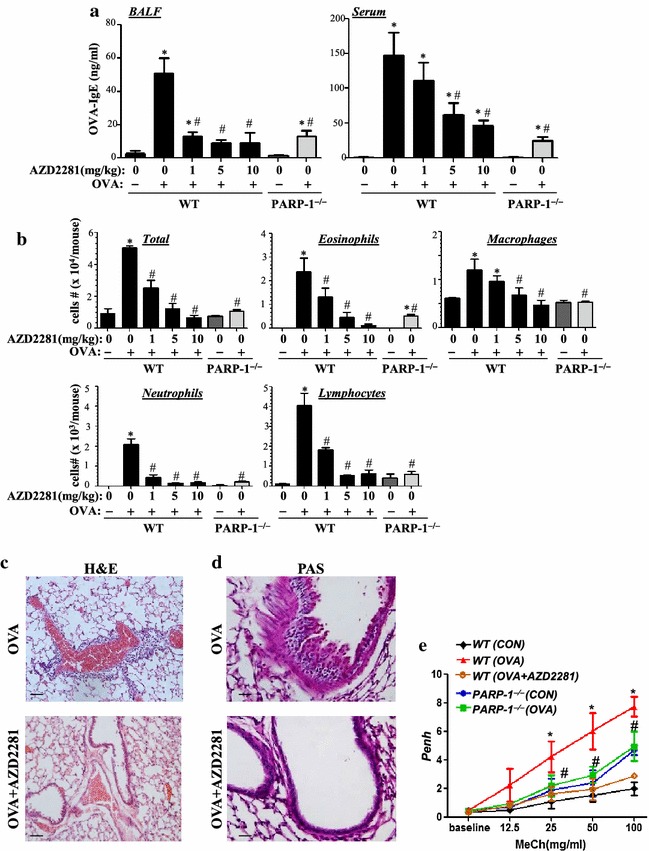
C57BL/6J WT or PARP-1^−/−^ mice were subjected to OVA sensitization followed by a single challenge to aerosolized OVA or left unchallenged. WT mice were administered, *i.p.*, 1 mg/kg, 5 mg/kg or 10 mg/kg of olaparib or saline thirty minutes after OVA challenge. Mice were sacrificed 48 h later and lungs were subjected to formalin fixation or BAL. **a** Assessment of BALF or sera collected from the different experimental groups 48 h after OVA challenge for OVA-specific IgE using sandwich ELISA. **b** Cells of BALF were differentially stained, and total cells, eosinophils, macrophages, lymphocytes, and neutrophils were counted. Data are expressed as total number of cells per mouse. Data are means ± SD of values from at least six mice per group. **c** Lung sections from OVA-challenged mice that were treated with either saline or olaparib were subjected to H&E or **d** PAS staining. **e** Mice were sensitized and challenged with OVA as described above. A group of WT mice received an injection of 5 mg/kg of olaparib. Penh was recorded 24 h later using a whole body plethysmograph system before and after the indicated concentrations of aerosolized methacholine (MeCh). Results are plotted as maximal fold increase of Penh relative to baseline and expressed as mean ± SEM where n = 6 mice per group. For **a**, **b**, and **e** *asterisk* difference from control unchallenged mice, *p* < 0.01; *hash* difference from OVA-challenged mice; *p* < 0.01. For **c** and **d** *bar* 5 μm.

Figure 1a shows that a single administration of olaparib at the 1 mg/kg dose almost completely prevented the elevation of OVA-specific IgE production in BAL fluids (BALF) but not sera collected from OVA-sensitized and challenged mice. A slightly higher dose of 5 mg/kg was sufficient to cause a significant reduction in the sera levels of OVA-specific IgE. As expected, PARP-1 gene deletion provided similar protection. The blockade in IgE production coincided with a significant reduction in the total number of inflammatory cells recruited to the lung of treated animals with a prominent effect on eosinophils, neutrophils, and lymphocytes (Figure 1b). Figure 1c shows an example of the inflammatory cell infiltration into the lungs of OVA-challenged mouse and the effective protection against such infiltration by treatment with 5 mg/kg olaparib as assessed by H&E staining. Treatment with olaparib also reduced mucus production as assessed by Periodic acid–Schiff (PAS) staining (Figure 1d). Figure 1e shows that administration of 5 mg/kg olaparib almost completely prevented AHR manifestation to increasing doses of methacholine. The effects of olaparib administration were similar to those observed in OVA-challenged PARP-1^−/−^ mice.

**Figure 2:**
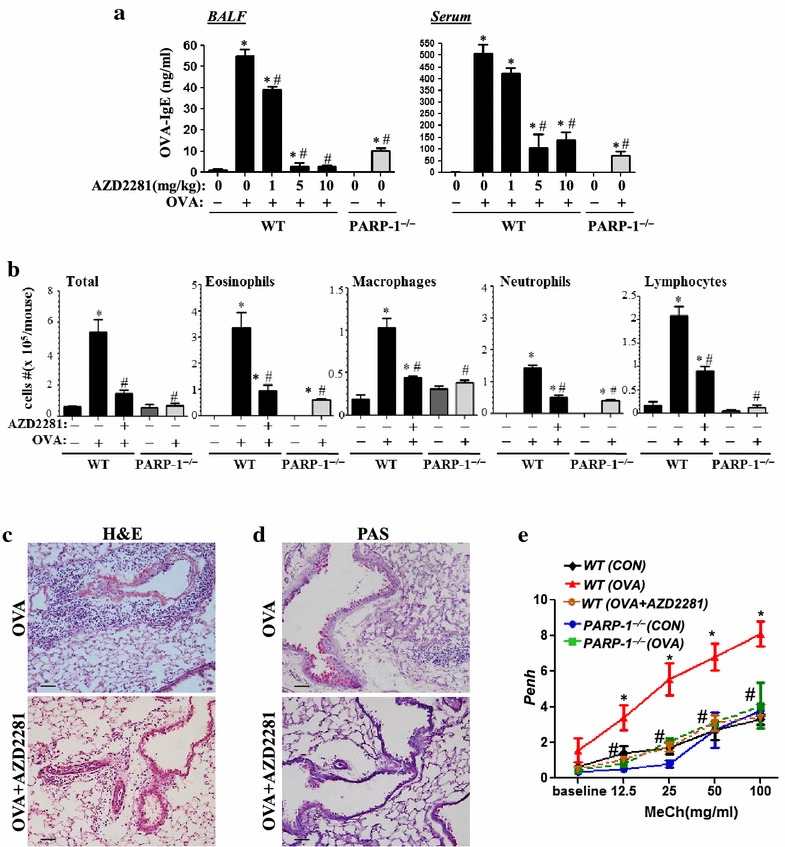
WT or PARP-1^−/−^ mice were subjected to OVA sensitization followed by triple challenge (Multiple) or left unchallenged. WT mice were administered, *i.p.*, 1 mg/kg, 5 mg/kg or 10 mg/kg of olaparib or saline thirty minutes after each challenge. Mice were sacrificed 48 h later and lungs were subjected to formalin fixation or BAL. **a** Assessment of BALF or sera collected from the different experimental groups 48 h after the last challenge for OVA-specific IgE. **b** Cells of BALF were differentially stained, and total cells, eosinophils, macrophages, lymphocytes, and neutrophils were counted. Data are means ± SD of values from at least six mice per group and are expressed as total number of cells per mouse. Lung sections from multiple OVA-challenged mice that were treated with either saline or olaparib were subjected to H&E **c** or PAS **d** staining. **e** Mice were sensitized and challenged with OVA as described above. A group of WT mice received an injection of 5 mg/kg of olaparib. Penh was recorded 24 h later using a whole body plethysmograph system before and after the indicated concentrations of aerosolized methacholine (MeCh). Results are plotted as maximal fold increase of Penh relative to baseline and expressed as mean ± SEM where n = 6 mice per group. For **a**, **b**, and ** e** *asterisk* difference from control unchallenged mice, *p* < 0.01; *hash* difference from OVA-challenged mice; *p* < 0.01. For **c** and **d** *bar* 5 μm.

The protective effect of olaparib against a single OVA challenge does not necessarily mean that the drug would maintain its anti-inflammatory efficacy upon multiple challenges. Accordingly, mice were challenged daily for three consecutive days and received increasing doses of olaparib 30 min after every challenge. Figure 2a shows that olaparib maintained a remarkable efficacy in reducing OVA-specific IgE production with a maximal protection conferred by the 5 mg/kg dose of the drug. At this dose, the drug exerted a pronounced protection against the inflammatory burden induced by repeated OVA challenges including eosinophilia (Figure 2b, c), mucus production (Figure 2d), and AHR (Figure 2e) in a manner similar to that conferred by PARP-1 gene deletion.

### Olaparib treatment differentially affects production of Th1 and Th2 cytokines

**Figure 3:**
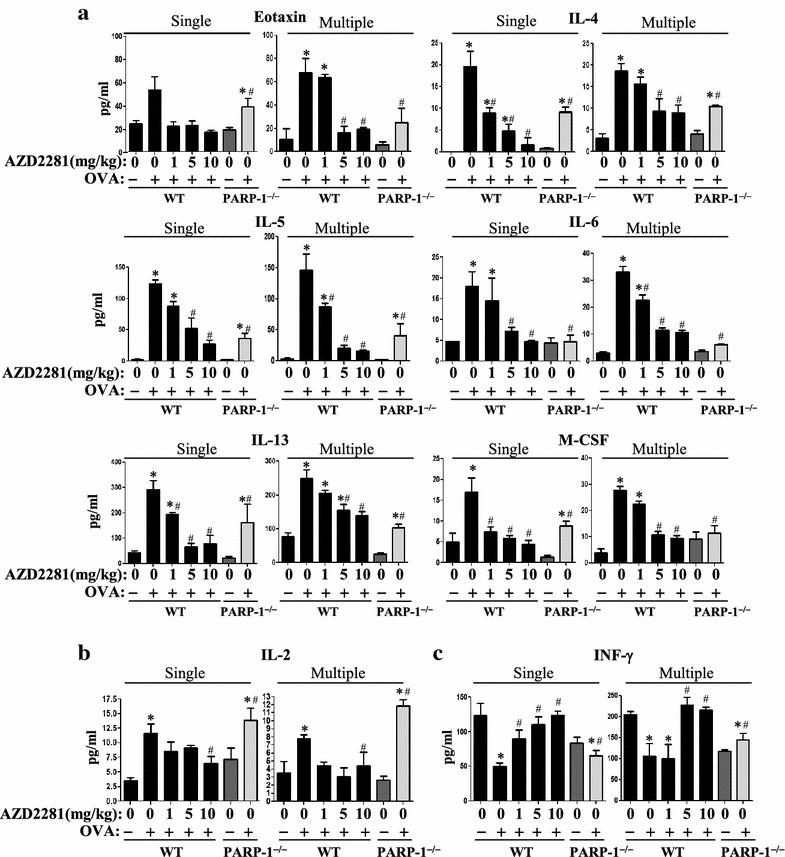
WT or PARP-1^−/−^ mice were subjected to OVA sensitization followed by a single or triple challenge (Multiple) or left unchallenged. WT mice were administered *i.p.* 1 mg/kg, 5 or 10 mg/kg of olaparib or saline 30 min after each challenge. Mice were sacrificed 48 h later and lungs were subjected to BAL. Assessment of BALF from the different groups for Th2 cytokines eotaxin, IL-4, IL-5, IL-6, IL-13, or M-CSF (**a**), IL-2 (**b**) or INF-γ (**c**). Data are means ± SD of values from at least six mice per group. *Asterisk* difference from control unchallenged mice, *p* < 0.01; *hash* difference from OVA-challenged mice; *p* < 0.01.

Figure 3a shows that both single and multiple OVA challenge induced considerable levels of several Th2 cytokines including eotaxin, IL-4, IL-5, IL-6, IL-13, and M-CSF, and that olaparib administration suppressed production of these cytokines. It is important to note that in the single OVA challenge model, olaparib at 1 mg/kg provided a remarkable reduction in the production of the aforementioned cytokines most notably eotaxin, IL-4, and M-CSF. Upon repeated OVA challenges, the lowest dose of olaparib only reduced the levels of IL-5 and IL-6. However, the 5 mg/kg dose was sufficient to almost completely block the production of all measured cytokines. It is worth mentioning that the effect of PARP inhibition either pharmacologically or by gene knockout on IL-2 production was marginal in both the single and repeated OVA challenge models (Figure 3b).

Figure 3c shows that the levels of IFN-γ were reduced upon a single or repeated challenge with OVA. Such decrease was prevented by administration of the PARP inhibitor. Interestingly, the levels of IFN-γ were markedly lower in control PARP-1^−/−^ mice and, unlike in olaparib-treated animals, OVA challenge did not cause an elevation of the cytokine in the knockout animals.

### PARP inhibition by olaparib or gene knockout prevents OVA challenge-induced elevation in CD4^+^ T cells but increases T-reg cell population in spleen of treated mice

**Figure 4:**
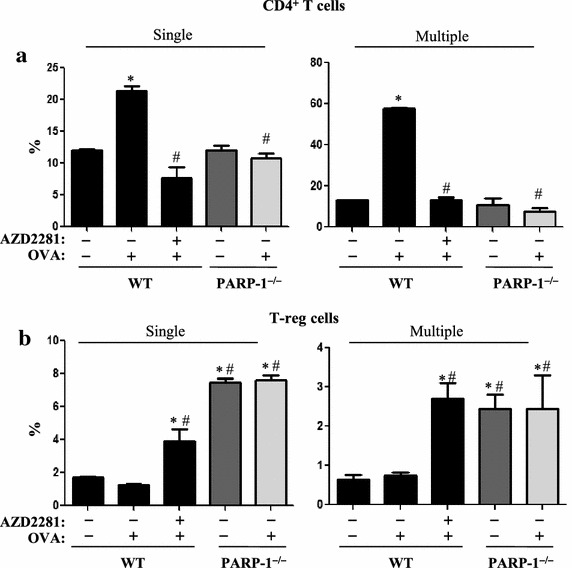
WT or PARP-1^−/−^ mice were subjected to OVA sensitization followed by a single or triple challenge (Multiple) or left unchallenged. WT mice were administered *i.p.* 5 mg/kg of olaparib or saline 30 min after each challenge or left untreated. All mice were sacrificed 48 h later. Spleens from the different experimental groups were used to generate single-cell suspensions. **a** Cells were stained with antibodies to CD3e (145-2c11-APC) and CD4 (GK1.5-PE). **b** A portion of the cells was also subjected to intracellular staining with antibodies to Foxp3 (FJK-16s- PE) in addition to antibodies to CD4 (GK1.5-FITC) and CD25 (PC61.5- APC). Stained cells were then analyzed by FACS. *Asterisk* difference from control WT mice, *p* < 0.01; *hash* difference from WT-OVA challenged mice, *p* < 0.01.

Given the substantial effect of PARP inhibition on Th2 cytokine production, we next examined whether PARP inhibition achieved such effect by modulating CD4^+^ T cell populations in spleens of OVA-challenged mice. PARP inhibition by olaparib or gene knockout did not cause any noticeable change in the overall number of cells in spleens of control or OVA-challenged mice (data not shown). However, PARP inhibition prevented the increase in the percentage of CD4^+^ T cells both upon a single (Figure 4a) or repeated (Figure 4b) OVA challenge. Conversely, the percentage of T-reg cell population increased upon olaparib administration. The T-reg cell population in naïve untreated PARP-1^−/−^ mice was higher than that in OVA-challenged WT mice. However, single or repeated OVA challenge did not culminate in an additional increase of such population in the mutant mice. It is unclear whether PARP inhibition-associated elevation in T-reg cell population was due to changes in the number of CD4^+^ T cells. However, these results suggest a potentially important role for PARP-1 in CD4^+^ T cell function.

### PARP inhibition by olaparib or gene knockout modulates CD4^+^ T cell function by differentially affecting expression of gata-3 and t-bet in CD3/CD28-treated CD4^+^ T cells

**Figure 5:**
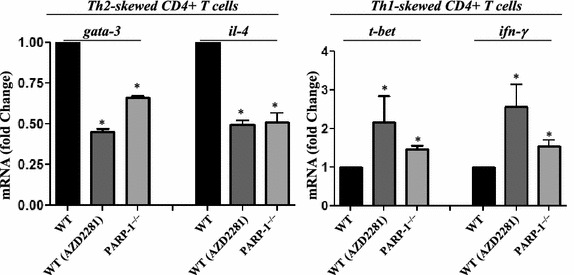
Purified CD4^+^ T cells procured from spleens of OVA-sensitized WT or PARP-1^−/−^ mice were stimulated with anti-CD3 and anti-CD28 antibodies and then skewed into a Th1 or Th2 phenotype in the presence or absence of 5 μΜ olaparib. RNA was extracted then used to generate corresponding cDNA followed by quantitative PCR with primer sets specific for mouse *gata*-*3*, *il*-*4*, *t*-*bet*, *ifn*-*γ*, or *β*-*actin*. Data is expressed as fold change with *β*-*actin* as a reference gene. *Asterisk* difference from CD3/CD28-stimulated cells; *p* < 0.01.

We next examined whether the effect of olaparib on Th2 and Th1 cytokines was by controlling mRNA expression of key transcription factors that regulate the expression of these cytokines focusing primarily on *gata*-*3,t*-*bet, IL*-*4,* and *IFN*-*γ*. To this end, CD4^+^ T cells were skewed toward a Th1 or Th2 phenotype and stimulated with anti-CD3/CD28 antibodies in the presence or absence of 5 μM olaparib. Figure 5 shows that olaparib markedly reduced CD3/CD28-stimulated GATA-3 mRNA expression with a concomitant reduction in IL-4 mRNA expression. Interestingly, olaparib treatment caused an elevation in T-bet and IFN-γ mRNA expression in Th1-skewed CD4^+^ T cells. These results are consistent with the effect of PARP inhibition on the Th1 and Th2 cytokines observed in the animal models.

### Adoptive transfer of in vitro Th2-skewed OT-II CD4^+^ T cells is sufficient to reverse airway inflammatory cell recruitment, Th2 cytokine production, and OVA-specific IgE secretion in OVA-exposed PARP-1^−/−^ mice

**Figure 6:**
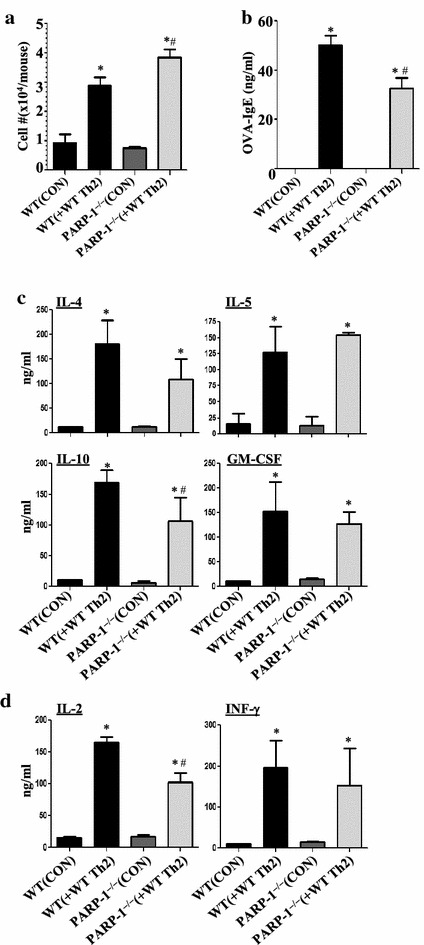
**a** OT-II mice were sacrificed and CD4^+^ T cells were skewed towards a Th2 phenotype in presence of the OT-II peptide. Cells (1 × 10^6^ per mouse) were injected *i.v.* into the tail vein of naïve WT or PARP-1^−/−^ recipient mice. All mice were subjected to aerosolized OVA challenge daily for 4 consecutive days. Forty-eight hours later, mice were sacrificed and subjected to BAL. BALF were subjected to total cell count. Sera were assessed for OVA-specific IgE (**b**). BALF were also assessed for the Th2 cytokines IL-4, IL-5, IL-10, and GM-CSF (**c**) or the Th1 cytokines IL-2 and INF-γ (**d**). *Asterisk* difference from control WT mice, *p* < 0.01; *hash* difference from WT mice receiving adoptive transfer (WT (+WT Th2), *p* < 0.01.

Overall, the above results suggest that PARP-1 not only plays a role in CD4^+^ T cell recruitment but also plays a critical role in the function of these cells. To test the role of PARP-1 in CD4^+^ T cell function during an allergic response, we examined whether adoptive transfer of WT CD4^+^ T cells isolated from OT-II mice that were skewed in vitro toward a Th2 phenotype reverses asthma-like traits in naïve PARP-1^−/−^ mice upon OVA exposure. Figure 6a shows that, indeed, transfer of Th2-skewed CD4^+^ T cells was sufficient to reverse lung inflammation. Such effect occurred concomitantly with an elevation in OVA-specific IgE production (Figure 6b) and production of the Th2 cytokines IL-4, IL-5, IL-10, and GM-CSF (Figure 6c) in addition to the Th1 cytokines IL-2 and IFN-γ (Figure 6d) in PARP-1^−/−^ mice upon exposure to aerosolized OVA to levels equivalent or close to those observed in the WT counterparts. These results clearly suggest a critical role for PARP-1 in the CD4^+^ T cell function.

## Discussion

In this study, we show that olaparib administration is highly efficient in blocking established AAI and AHR, which constitute two major components of asthma. We also provide evidence for an important role for PARP-1 in CD4^+^ T cell function without a prominent effect on B cell function. Moreover, our results support the possibility that PARP inhibition may also influence T-reg cell accumulation as an additional mechanism in dampening allergic response in our experimental models. Lastly, the effect of olaparib on CD4^+^ T cell function may be strongly linked to the ability of PARP-1 to control expression of the transcription factor GATA-3.

Olaparib treatment was very effective in blocking repeated challenges to OVA in mice. Remarkably, a dose as low as 1 mg/kg of the PARP inhibitor was sufficient to confer protection against the manifestation of several asthma-like traits including AHR. As very recently shown by one of us [26], olaparib is also effective in reducing lung inflammation induced by LPS and inhibits expression of several inflammatory factors including VCAM-1 and TNF-α. Our results show that a major role of PARP may be in the function of CD4^+^ T cells. This is supported by the finding that an adoptive transfer of OT-II CD4^+^ T cells was sufficient to reverse lung cellularity and production of Th2 cytokines and IgE to levels comparable to those detected in similarly treated WT mice. The Th1 cytokines were also elevated. An increase in IL-2 is expected as it is critical for CD4^+^ T activation [27]. However, the production in IFN-γ was surprising. Although speculative, it is possible that the increase in IFN-γ was mediated by PARP-1^−/−^ CD4^+^ T cells in the presence of IL-2 produced by the adoptively transferred WT CD4^+^ T cells. It is also possible that the increase in IFN-γ may be mediated by PARP-2. This is based on the observation that PARP-1 gene knockout only slightly increase the expression of the Th1 cytokine while treatment with olaparib, which inhibits both PARP-1 and PARP-2, substantially increased it (Figure 3c). It is important to acknowledge that the study, as conducted, does not cover all the aspects of asthma manifestation and it remains to be determined whether the transfer of WT CD4^+^ T cells is sufficient to reverse AHR and mucus production in PARP-1^−/−^ mice. Although more specific experimentation is required, it is tempting to conclude from the adoptive transfer study that PARP-1 may not play a direct role in B cell function. The adoptive transfer of OT-II CD4^+^ T cells was sufficient to induce substantial levels of OVA-specific IgE. Such immunoglobulin production could have only been produced by PARP-1^−/−^ B cells clearly suggesting that the function of these cells is comparable to that of WT B cells in response to OVA challenge. We speculated in our previous studies that the primary reason for the reduced production of IgE upon PARP inhibition is the effect on IL-4 production [5, 6]. We cannot, however, exclude the possibility that PARP plays a role in B cell trafficking especially when considering the effect of PARP inhibition, pharmacologically or by gene knockout, on the overall recruitment of lymphocytes to the lung as shown in Figures 1b and 2b. The role of PARP-1 in GATA-3 expression may be the driving cause for the ability of PARP inhibition to reduce IL-4, IL-5, and IL-13 production. It is noteworthy that GATA-3 is the master regulator for the development of Th2 cells [28] through its ability to control the activation of the *Il4/Il5/Il13* cytokine locus.

The role of PARP-1 in T-reg cell accumulation has been reported in mice, which was associated with an increase in Foxp3 [29]. We confirm these results in the experimental AAI setting. Although olaparib increased the T-reg (CD4^+^/CD25^+^/Foxp3^+^) cells upon a single or repeated OVA challenge, T-reg cells were increased in PARP-1^−/−^ mice regardless of challenge with OVA. This suggests that PARP-1 moderately regulates T-reg cells but not upon an inflammatory response. Whether the slight increase in T-reg cells is a major driving force in the anti-inflammatory effect of PARP inhibition is not clear. Interestingly, a recent study demonstrated that T-reg cells isolated from PARP-1^−/−^ mice are as functional as those isolated from WT mice [30]. Overall, the present studies provide critical information on the role of PARP-1 upon an acute or established AAI and AHR and provide support to the notion that PARP can be targeted for the treatment of some aspects of human asthma.

Almost two dozen clinical trials most of which are in phase II or III are currently examining the possibility of establishing olaparib as a mono or adjuvant therapy for some specific cancers with BRCA mutation [19]. It is noteworthy that there are additional drugs with varying potency in inhibiting PARP under clinical trials most of which focus on the synthetic lethality induced by the drugs in BRCA-mutant cancer cells [19]. This phenomenon, as stated above, spares normal cells while targeting specifically the mutant cancer cells leading to their demise as a result of the accumulation of a fatal level of dsDNA breaks. It is important to note that the overarching assumption of these clinical trials is that these drugs do not have any important negative effects on normal cells and tissues. According to a clinical trial conducted by Fong et al. [21], a total of 200 mg olaparib, daily for more than 24 weeks, did not cause any side effects. This dose represents a 2.3 mg/kg for men with an average weight of 87 and 2.69 mg/kg for women with an average weight of 74.4. These doses fall between the 1 and 5 mg/kg doses used in the current study with which we observed substantial protection against experimental asthma. It is important to note that in the aforementioned clinical study and others [31–33] on higher doses of olaparib for patients with breast or ovarian cancer, the most common side effects were nausea, vomiting, fatigue and anemia. Despite these effects, discontinuation of the drug due to these side effects was a rare event. Additionally, patients with advanced cancer may be more prone to adverse events than asthma patients. Nevertheless, this would need to be tested closely in any human clinical study. The likely reduced side effects associated with the use of low doses of olaparib or other PARP inhibitors is very promising for the potential use of these drugs in treatment regimens against human asthma. Furthermore, treatment regimens may be extensive and lengthy in cancer, which may not be the case in asthma predicting that the use of olaparib in asthma may be associated with lesser side effects. Perhaps the therapeutic potential of olaparib may become more relevant to difficult to treat asthma especially those that do not respond to corticosteroids. Although we remain cautious, our study suggests that olaparib and potentially other PARP inhibitors are ready for testing on human asthma.

## Conclusion

Overall, the results of the present study provide more support for the role of PARP-1 in asthma pathogenesis and the potential of PARP inhibition as a viable therapeutic strategy for the treatment of asthma in humans. More importantly, our results propose olaparib as a likely candidate to be tested in human asthma clinical trials.

## Abbreviations

AAI: Allergic airway inflammation
AHR: airway hyperresponsiveness
BAL: bronchoalveolar lavage
BALF: BAL fluids
dsDNA: double-stranded DNA
OVA: ovalbumin
PARP-1: Poly(ADP-ribose)polymerase-1
PAS: Periodic acid–Schiff
T-reg: CD4^+^ T regulatory cells
